# The validity of Engagement and Feedback Assessments (EFAs): identifying students at risk of failing

**DOI:** 10.1186/s12909-023-04828-7

**Published:** 2023-11-15

**Authors:** Paul R. Kemp, Jacob M. Bradshaw, Brijmohan Pandya, Daniel Davies, Mary J. Morrell, Amir H. Sam

**Affiliations:** 1 Imperial College School of Medicine, Imperial College London, London, UK; 2 National Heart and Lung Institute, Imperial College London, London, UK

**Keywords:** Assessment, Attendance, Medical students, Predicting failure, Engagement and feedback assessments, Kane’s validity framework, Validity

## Abstract

**Background:**

Imperial College School of Medicine, London UK, introduced a new curriculum in 2019, with a focus on the GMC outcomes for graduates, and pedagogy best practice. The new curriculum included formative assessments, named engagement and feedback assessments (EFAs), to support learning, and attainment in the summative examinations. The aims of this study were to assess the validity of EFAs and to determine whether they have utility as a modified form of programmatic assessment to inform decision-making regarding possible interventions by measuring and analysing attendance at and performance in these formative events.

**Methods:**

Seven hundred and sixty-one students were included in the study and assessment results were included for academic years 2019/20 to 2020/21. Forty-one data points per student, (27 in Year 1 and 14 in Year 2) were used, to compare EFA scores with the summative performance. Attendance was monitored through engagement with the EFAs.

**Results:**

Cohort 1 (enrolled 2019): In year 1, EFAs were associated with summative exam scores (overall *r* = 0.63, *p* < 0.001). Year 2, EFA scores were also associated with summative scores (overall *r* = 0.57, *p* < 0.001), including the clinical practical assessment (*r* = 0.45, *p* < 0.001).

Missing two or more EFAs was associated with a significant increase in the likelihood of failing one or more summative examinations in the first year (OR: 7.97, 95% CI 2.65–34.39) and second year (OR: 3.20, 95% CI 1.74–5.95). Missing more than two EFAs in their first year was also associated with a higher risk of failing a summative examination in the second year (OR: 2.47, 95% CI 1.33–4.71). Students who increased their attendance between year 1 and 2 fared better in summative assessment than those who maintained poor attendance, whereas those that reduced their attendance fared worse than those that maintained high attendance.

Cohort 2 (enrolled 2020): Analysis of cohort 2 supported these findings and in this cohort missing two or more EFAs was again associated with an increased likelihood of failing a summative examination (OR = 4.00, 95% CI = 2.02–7.90).

**Conclusion:**

Our EFA model has validity in predicting performance in summative assessments and can inform prospective interventions to support students’ learning. Enhancing attendance and engagement can improve outcomes.

**Supplementary Information:**

The online version contains supplementary material available at 10.1186/s12909-023-04828-7.

## Background

Learning requires an appropriate level of prior understanding, cognitive function, and engagement by the student with the course material. Monitoring engagement is difficult in the university setting as students begin more self-directed learning and the interaction time with teachers becomes more flexible. One often-used measurement of engagement is attendance at teaching events. The ability of in-class attendance to predict assessment outcomes in medical students is not clear, with some studies showing little association [1–4] and others showing positive associations with increased attendance [5–10] and negative association with decreased attendance [11]. In a large meta-analysis amongst US undergraduate college students, the association between attendance and grades was found to be moderately strong [12]. The disparity in findings has led to some doubt about the importance of attendance. Furthermore, attendance and engagement are not the same things with some students being ‘present but not involved’ thus crude attendance statistics may not provide a complete picture. Parallel work on professionalism has shown that the collection of routine measures of attendance, combined with engagement may better predict students who are likely to be lower-performing students in medical school and their early clinical practice [13–15].

The self-directed learning often required at university means that the approaches students use to learn may need to be very different from those employed within a school environment. A student’s own ability to develop their learning techniques within this environment and to understand their performance is key to them achieving their potential within the parameters of the course [16]. In medical education, it has been suggested that developing students’ understanding of how they perform is an important part of developing an ability to monitor their own decisions in clinical practice [17]. However, many students do not use the best methods of learning and are unable to properly monitor their progress [16].

Teaching students how to become active learners and how to monitor their progress has the potential to improve overall learning. Within a modern medical school, there are at least 2 significant issues affecting incorporating the teaching of the science of learning successfully. The first impediment is the time within the curriculum needed to give students the appropriate information which will undoubtedly take time away from areas that members of faculty deem to be more important [18]. The second and probably most difficult is that students starting medical schools in the UK have used learning strategies that have been successful enough to get them to medical school [18]. However, these may have been used within a more structured environment and may not adapt well to the independent learning that they need at university.

It is therefore important to identify students who are at risk of failing the course, and various factors can be used to achieve this, including pre-entrance assessment results, completion of routine tasks, and attendance and learning analytics [19–23].

Regular short engagement and feedback assessments (EFAs), were introduced at Imperial College School of Medicine drawing on the principles of programmatic assessment [24] where the assessment is formative but the students sit pass/fail summative assessments [25]. This allows a form of programmatic assessment in cases where regulatory constraints prevent utilisation of the full programmatic approach to progression. The purpose of these EFAs was to inform students of their progression and drive continuous engagement, as well as assess whether they could assist Faculty in the early identification of students experiencing difficulties to provide an opportunity for early intervention. Previous literature has shown that measures of engagement can identify students at higher risk of failing examinations [22]. Each EFA covered the material taught in the preceding two weeks. Incorporating principles of programmatic assessment in this format allowed for individualised feedback through multiple data points [26].

The validity of EFAs for utilisation as a predictive tool will be assessed in this study utilising Kane’s validity framework, which is an argument consisting of four separate components; scoring, generalisation, extrapolation, and implications [27]. The scoring component regards the individual items and response options, as well as the scoring rubric and item analysis. EFAs consist of Single Best Answer Questions (SBAs), as well as Very Short Answer Questions (VSAQs), and a single Short Answer Question (SAQ). It has been discussed that the use of multiple assessment instruments in assessment constructs provides a more complete picture [28, 29]. In addition, previous studies have demonstrated that VSAQs have higher reliability, validity, and authenticity than SBAs, hence their inclusion within the EFAs [30–32].

To evaluate the other inferences of Kane’s framework, student performance in their summative examinations was compared with attendance at and performance in EFAs across two year cohorts of medical students enrolled at Imperial College School of Medicine. The primary aim of this study was to assess the validity of EFAs using Kane’s argument, to evaluate whether EFAs have utility as a modified form of programmatic assessment, which can be used to inform decision-making regarding possible interventions. One of the secondary aims was to determine if there was an association between students’ attendance and their summative examination score and whether students who changed their attendance between years changed their performance in summative assessments, to assess the potential implications of the validity framework. In addition, the second cohort was analysed separately to assess for reproducibility.

## Methods

### Structure of the ICSM new curriculum

Imperial College School of Medicine introduced a new curriculum for the academic year 2019/2020 in line with a College wide learning and teaching strategy and the General Medical Council outcomes for graduates [33]. A curriculum review had determined that a module-based integrated approach to learning, with a technology-enhanced, socially accountable, and evidence-based curriculum would future proof graduates for an uncertain and evolving future of the clinician’s role. Within this new curriculum a focus was placed on team-based learning events both as formative teaching and summative assessment [34].

**Fig. 1:**
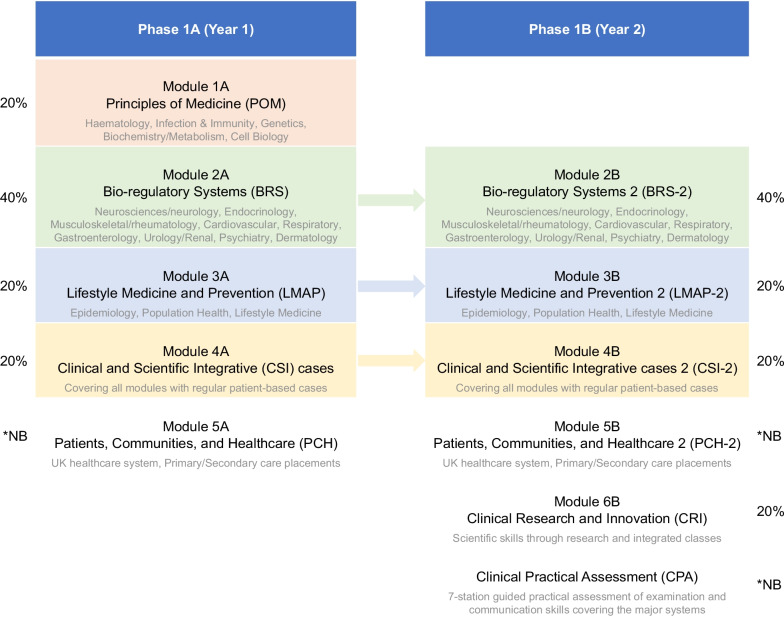
Curriculum map of Phase 1A (Year 1) and Phase 1B (Year 2) of the Medicine MBBS Programme at Imperial College School of Medicine. POM is limited to only Phase 1A and CRI is limited to only Phase 1B, whilst the other modules are continued from Phase 1A to 1B. The percentages represent the contribution of that module to the overall summative mark for the year. *NB: These modules are pass/fail progression, but the mark does not contribute to the overall weighted score for the year

The ICSM new curriculum has 5 modules in the first year, called Phase 1a, [A](Principles of Medicine [Module 1A], Bio-regulatory systems-1 [Module 2A], Lifestyle Medicine and Prevention-1 [Module 3A], Clinical and Scientific Integrative (CSI) cases -1 [Module 4A], and Patients Communities and Healthcare-1 [Module 5A]) and 5 modules in the second year, called Phase 1b [B], using a spiral curriculum approach (Bio-regulatory systems-2 [Module 2B], Lifestyle Medicine and Prevention-2 [Module 3B], Clinical and Scientific Integrative (CSI) cases -2 [Module 4B], Patients Communities and Healthcare-2 [Module 5B] and Clinical Research and Innovation[Module 6B]). See Fig. 1 for a curriculum map, including the percentage contribution of each module to the overall summative score, which are based upon teaching time dedicated to each module.

### Student cohorts

Cohort 1: 358 students enrolled in 2019 and completed year 1 (Phase 1a) in 2020 and year 2 (Phase 1b) in 2021. 355 students sat exams for Module 1A and 2A in April/May 2020 and 352 sat the exams for Module 2B and 3B April/May 2021. All written papers had to be passed independently.

Cohort 2: 403 students were enrolled in 2020 and completed Year 1 in 2021.

### Summative assessments

Each module was assessed independently using a range of different tools. Modules 1A, 2A, 2B and 3B were examined using a mixture of single best answer questions (SBAs), very short answer questions (VSAs) and short answer questions (SAQs). Modules 4A and 4B were assessed using team-based learning (TBL). Each TBL consisted of a 10 SBA individual Readiness Assurance Test (iRAT), with the same questions answered in a team RAT (tRAT) plus a team application test, which varied, to assess the learning outcomes. Modules 5A and 5B were assessed by workplace-based assessments. In Phase 1B the students also sit a Clinical Practical Assessment (CPA) to assess their clinical skills. Module 3A was examined using a podcast and Module 6B using a team poster presentation that will not be discussed further here. Additionally, the anatomy and diagnostics domain (located within module 2A) was assessed using a spotter exam consisting of anatomical images and associated VSAs.

### Structure and timing of EFAs

EFAs consisted of two components. Firstly, an Integrated Formative Test (IFT) consisting of 5 SBAs and 5 VSAs performed in class, which were marked soon after the event, and a structured SAQ, answered in the students’ own time. The answer to the SAQ was released after 1 week for the students to self-mark. The second component was Team Based Learning exercises (TBLs) in Modules 1A, 2A and 2B, consisting of 10 SBA iRAT and tRAT assessments analogous to the summative format.

Year 1 students (Phase 1a) also had two formative exams, with one covering learning from Modules 1A and 2A, (a mixture of SBA, VSA and SAQs) and an anatomy spotter (anatomical images and VSAs).

During Phase 1a, term 1 there were 3 IFTs and 7 TBLs for Module 1A (i.e., 10 EFA sessions) together with 1 EFA and 3 summative TBLs for Module 4A. During term 2 there were 3 IFTs and 4 TBLs for Module 2A together with 4 summative TBLs for Module 4A and 2 formative exams covering Modules 1A and 2A. Consequently, at the end of term 1, there were 14 data points and at the end of term 2 a further 13 data points.

During Phase 1b, term 1 there were 4 IFTs for Module 2B and 4 summative TBLs for Module 4B. During term 2 there were 3 IFTs for Module 2B and 3 summative TBLs.

### Standard setting

All summative examinations are set using the Ebel method. These are checked against modified Cohen for evaluation purposes. The CPA is a practical assessment and is set using borderline regression applied to each station and session separately. The EFAs (IFTs, TBLs) do not have a pass mark and are purely a source of information to guide potential intervention.

### Statistical analysis

Data were analysed by two separate researchers. Firstly, in Aabel 3.0, then corroborated in R statistical software. Data were assessed for normality using Shapiro–Wilk tests and, since all comparisons involved at least one non-normal distribution, non-parametric tests were used for further analysis. Overall correlations were calculated using Spearman rank. Differences between groups were calculated using the Mann–Whitney U test for 2 groups, the Kruskal–Wallis test for multiple groups, and pairwise comparisons using the Wilcoxon test with correction for multiple testing. Logistic regression models were used to predict whether attendance or performance in EFAs could affect the odds of failing at least one exam, with p values calculated from the likelihood ratio test.

## Results

The results for each diet of summative exams are shown in Table 1.

**Table 1:** Assessment performance for the two cohorts

Cohort	Phase	Assessment	No. of students	Average score	Max. score	Min. score	No. of fails (%)
1	1a	Module 1A	354	74.40	92.25	49.25	3 (0.85)
		Module 2A	355	77.16	93.39	55.50	0 (0.00)
		Spotter	355	76.67	100.00	10.20	17 (4.79)
	1b	Module 2B	355	68.45	88.33	33.33	28 (7.89)
		Module 3B	355	70.77	91.00	45.00	23 (6.48)
		CPA	352	81.94	96.73	49.02	16 (4.55)
2	1a	Module 1A	400	64.70	90.42	36.17	48 (12.00)
		Module 2A	400	72.27	88.47	42.22	6 (1.50)
		Spotter	400	75.76	97.56	29.27	39 (9.75)

### Does EFA performance associate with summative performance?

#### Cohort 1

**Fig. 2:**
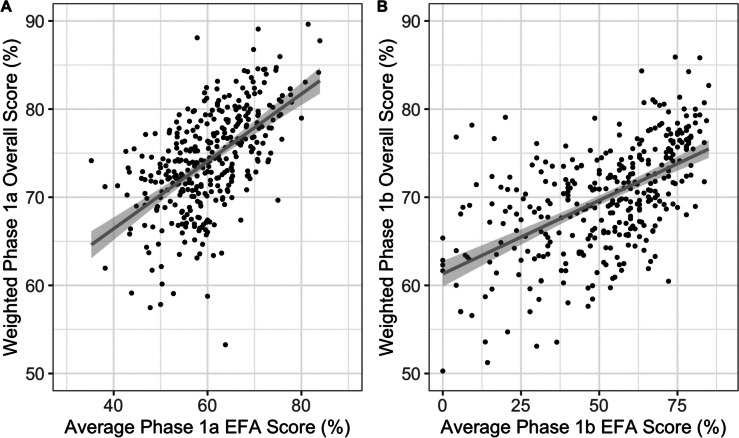
EFA performance was associated with summative exam performance in Cohort 1 across Phase 1a (**A**) and 1b (**B**). **A** shows that the average score for all EFAs attended in Phase 1a was positively correlated with exam performance for the cohort in the weighted overall score (*r* = 0.58, *p* < 0.001). **B** shows that the average score for all EFAs attended in Phase 1b was positively correlated with exam performance for the cohort in the weighted overall score (*r* = 0.58, *p* < 0.001)

To determine whether the programmatic EFA data was predictive of summative performance in year 1, the results from the EFA portfolio were compared with the Module 1A, 2A and anatomy spotter exams. Average EFA performance (the mean performance in all EFA sessions attended over 2 terms) was associated with overall summative performance (*r* = 0.58, *p* < 0.001, Table 2 and Fig. 2A). In addition, correlations with the individual summative component exams (Modules 1A, 2A and Spotter) were highly significant (Supplementary Table S1 and Supplementary Figure S1). Early EFA performance (in the first 6 weeks), also correlated strongly with overall summative performance (*r* = 0.53, *p* < 0.001, Table 2), as well as individual exams (Supplementary Table S1).

**Table 2:** Correlation of EFA results with overall summative results in Phase 1a and 1b

Phase	EFAs used	Correlation, r^a^	*p*-value^‡^
1a (Year 1)	Terms 1 and 2	0.58	< 0.001
	Term 1 only	0.53	< 0.001
1b (Year 2)	Terms 1 and 2	0.58	< 0.001
	Term 1 only	0.57	< 0.001

^a^Calculated using the Spearman rank correlation coefficient, $\rho$

^‡^This represents the first term only (i.e., very early performance

Comparison of Phase 1b EFA scores with Phase 1b summative performance showed similar associations not only with overall performance (*r* = 0.58, *p* < 0.001, Fig. 2B) and the Module 2B and 3B exams but also with performance in the clinical practical assessment, a seven-station assessment of clinical skills and associated knowledge (Supplementary Table 1 and Supplementary Figure S2). Similar to the pattern seen in Phase 1a, early EFA performance in Term 1 correlated with overall summative performance (*r* = 0.57, *p* < 0.001, Table 2), as well as with individual component exams (Supplementary Table S1).

An additional analysis was performed on early summative performance in Module 4A iRAT scores to determine whether early summative scores would correlate more strongly than early EFA scores with final summative scores. This did not reveal stronger association (Supplementary Results).

To determine if performance in early EFAs could predict failing exams, logistic regression models compared the top 50% versus bottom 50% of performers in EFAs and compared their odds of failing at least one summative exam. In Phase 1a, performance in TBLs in term 1 was not associated with increased odds of failing an exam (OR 1.28, 95% CI 0.54–3.05, *p* = 0.57). However, performance in term 1 IFT assessments in Phase 1a was associated with an increased odds of failing at least one summative assessment (OR 3.59, 95% CI 1.18–10.89, *p* = 0.024). Performance in term 1 Module 4A summative cases was also associated with increased odds of failing an exam (OR 3.09, 95% CI 1.11–8.56, *p* = 0.019).

As with Phase 1a, logistic regression models compared performance in term 1 EFAs with performance in Phase 1b. Being in the bottom half of performers in term 1 IFTs was associated with nearly 9 times the odds of failing at least one summative 1b exam (OR 8.92, 95% CI 3.67–21.72, *p* < 0.001). Performance in term 1 Module 4A summative exams was also associated with increased odds of failing an exam (OR 4.88, 95% CI 2.36–10.12, *p* < 0.001).

#### Cohort 2

To determine the robustness of our observations, we repeated the analysis using data from the cohort of students starting in 2020. These students had the same EFAs and summative assessments throughout the first 2 terms as cohort 1 with the exception that much of the delivery was remote and that all assessments were planned as open-book assessments, whereas exams for cohort 1 were planned as closed book but delivered open book due to the COVID-19 pandemic. Again, the EFA assessments were associated with summative exam performance (Module 1A: *r* = 0.56, *p* < 0.001, Module 2A main: *r* = 0.59 *p* < 0.001, Anatomy spotter: *r* = 0.51, *p* < 0.001). Similarly, autumn term EFA performance and the combination of all assessments (EFA and summative Module 4A TBLs) showed strong associations with summative performance (first term EFA vs Module 1: *r* = 0.56, *p* < 0.001, Module 2A main: *r* = 0.57 *p* < 0.001, Anatomy spotter: *r* = 0.47, *p* < 0.001 and first term all assessments Module 1: *r* = 0.60, *p* < 0.001, Module 2A main: *r* = 0.63 *p* < 0.001, Anatomy spotter: *r* = 0.49, *p* < 0.001). These data replicated the findings from cohort 1, showing that early performance in EFA assessments associated strongly with later summative performance and that the more assessments included, the stronger the associations.

### Does attendance at EFAs associate with summative score or likelihood of failing an exam?

We predicted that attendance would be a key metric in quantifying engagement with the course and therefore likely success. Given that some absences will be justified on health or similar grounds we divided the cohorts into those who had missed 0 or 1 EFAs and/or Module 4A/B summative events and those who had missed 2 or more. The mean score in each assessment and the likelihood of failing at least one exam was determined for each group.

#### Cohort 1

**Fig. 3:**
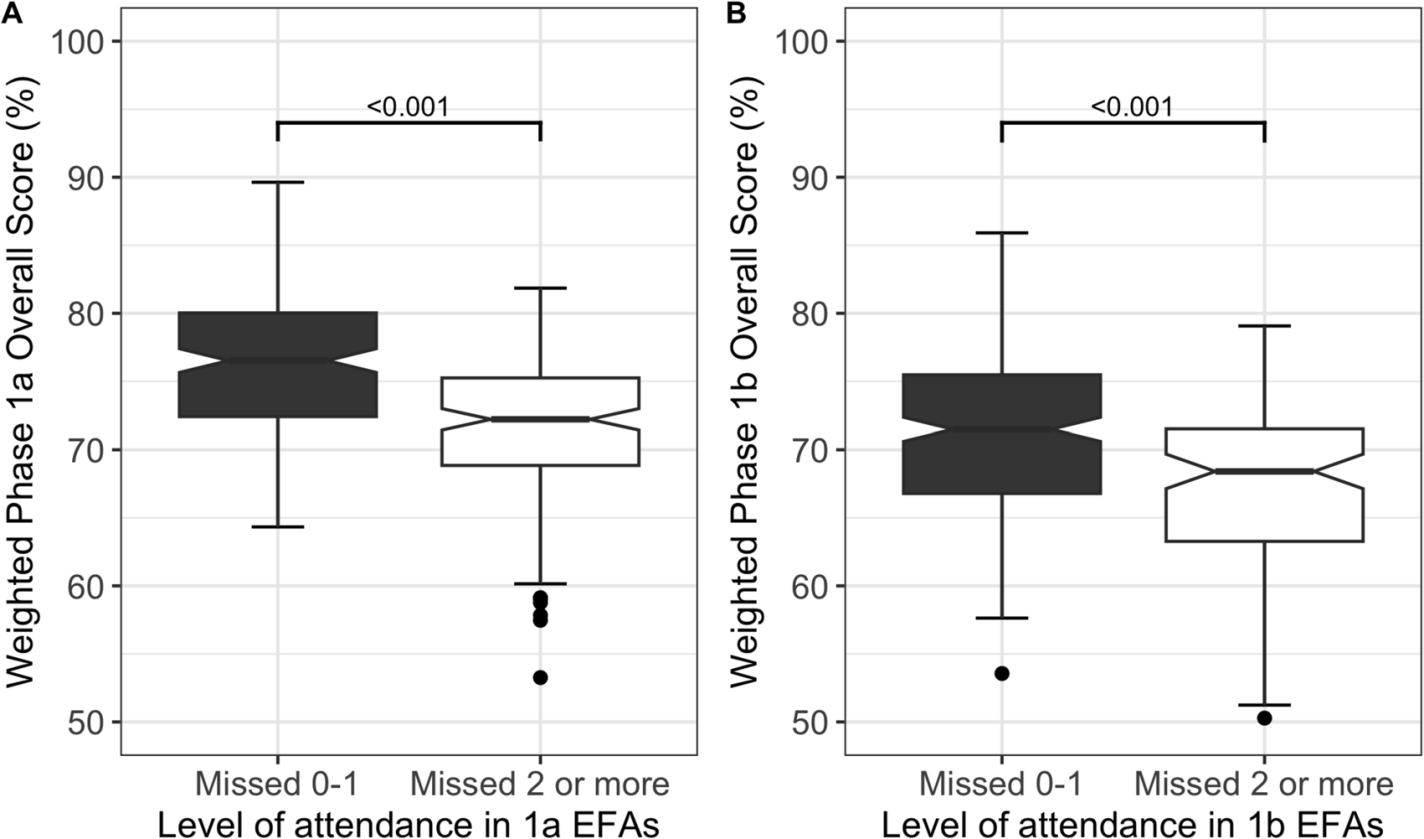
Effect of missing more than 2 events on the score for cohort 1 in Phase 1a (**A**) and Phase 1b (**B**). Students were grouped by attendance at formative events into those who missed 0 or 1 event and those who missed 2 or more events. **A** shows that students who had the greatest attendance performed better than those with lower attendance in cohort 1 for Phase 1a (*P* < 0.001 MW). **B** shows that students who had a higher attendance outperformed those with lower attendance for the whole of cohort 1 in Phase 1b (*P* < 0.001 MW)

In all summative assessments, missing two or more EFAs was associated with a reduction in the median score of at least 4% (from 8.4% for the Anatomy spotter to 4.0% for the Module 2A paper) and these were all statistically significant (*p* < 0.001 Mann–Whitney, Supplementary Figure S3). The overall weighted exam score was 4.36% (3.25 to 5.46) lower (Fig. 3A). Furthermore, in the group who missed two or more sessions, 19/166 students failed at least one paper with one student failing two papers, whereas only 3/188 students failed any paper in the rest of the cohort. Missing two or more sessions was associated with nearly an eightfold increase in the odds of failing one or more papers (OR: 7.97, 95% CI 2.31–27.45, *p* < 0.001, logistic regression). Interestingly this trend continued and students missing 2 or more EFA sessions in Phase 1a were also more likely to fail at least 1 Phase 1b paper but the effect was weaker (OR: 2.47, 95% CI 1.32–4.62, *p* = 0.004).

The same pattern of results was seen for cohort 1 in Phase 1b. Students missing two or more sessions (Fig. 3B) scored lower by between 3 and 8% in their summative papers (Supplementary Figure S4). Missing two or more Phase 1b sessions was associated with a marked increase in the likelihood of failing a Phase 1b paper (OR: 3.20, 95% CI 1.74–5.90, *p* < 0.001).

Students who missed two or more EFA sessions in Phase 1a were also more likely to miss multiple EFA sessions in Phase 1b than students who missed 1 or fewer sessions (OR: 5.61, 95% CI 3.37–9.34, *p* < 0.001).

#### Cohort 2

A similar analysis in cohort 2 gave the same findings with students missing two or more sessions scoring between 3 and 10% less in their summative with a reduction in overall assessment score of 4.46 (2.51 to 6.40, Supplementary Figure S5). In this diet of exams, student who missed two or more sessions in Phase 1a had 4 times the odds of failing at least one summative paper (OR = 4.00, 95% CI = 2.01–7.90).

### Does the performance of students whose attendance altered between years change?

The association between attendance and exam performance raises the possibility that students who alter their attendance behaviour between the two years would see a relative change in performance with those increasing their attendance improving and those reducing their attendance performing worse. As the absolute number of fails was insufficient to use this endpoint as a metric, we compared the performance of students in the Module 2A and Module 2B exams because this module is taught in a spiral manner across two years. Students were divided into four groups: group 1 missed 0 or 1 EFA exams in Phase 1a, and 0 or 1 in Phase 1b (continued high attendance in Phase 1b); group 2 missed 0 or 1 events in Phase 1a and 2 or more in 1b (declined attendance in 1b); group 3 missed 2 or more in Phase 1a and 2 or more in 1b (continued poor attendance in 1b); and group 4 missed 2 or more in Phase 1a and 0 or 1 in 1b (improved attendance in 1b).

**Fig. 4:**
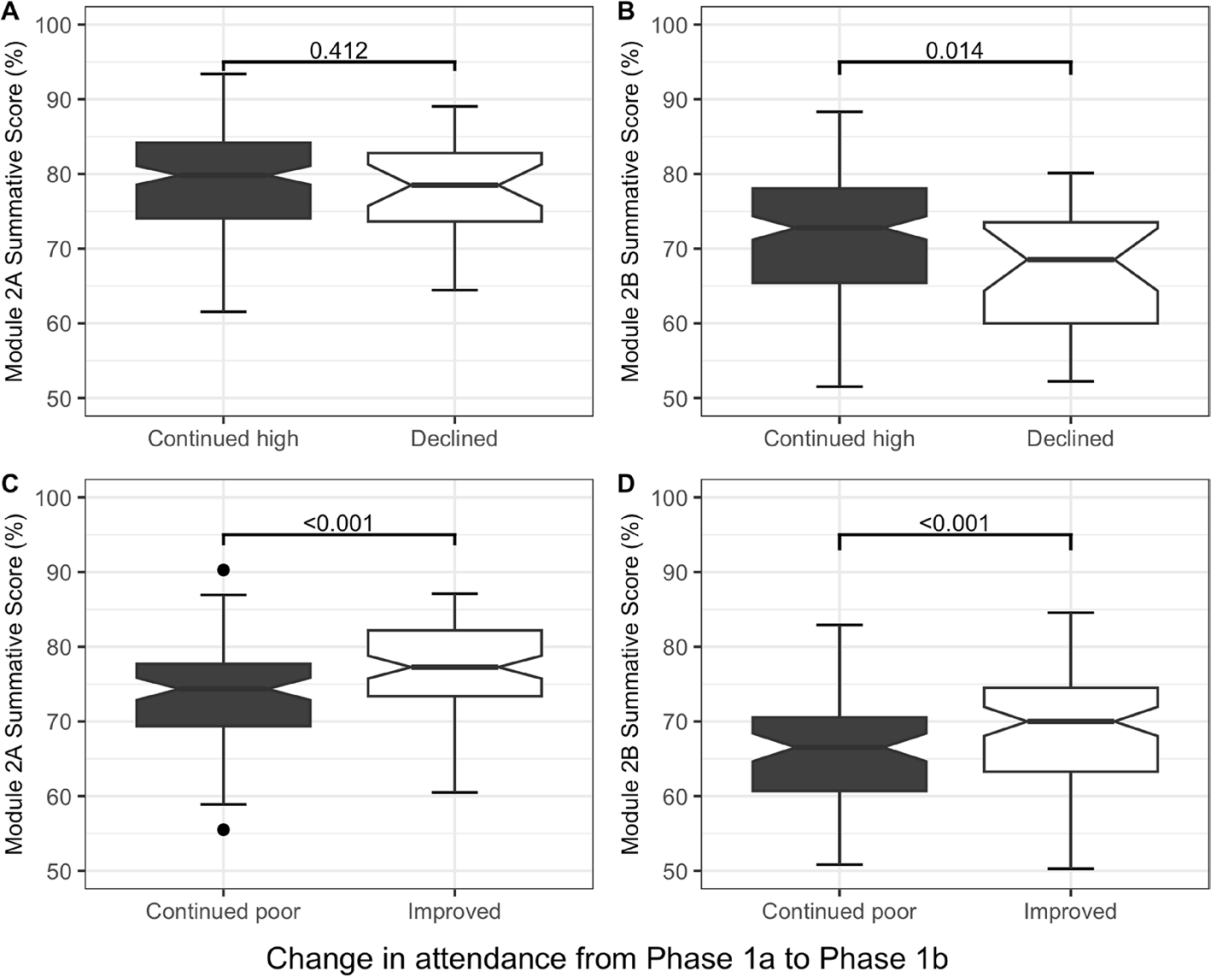
Effect of changes in attendance behaviour on exam score. Exam scores from Module 2A (Phase 1a) and Module 2B (Phase 1b) can be seen. Students were divided into 4 groups based on attendance as described above. **A** shows that those with continued high attendance (group 1) had similar scores to those with declined attendance (group 2) in Module 2A in Phase 1a (*p* = 0.41), whilst **B** shows that they scored better in Module 2B in Phase 1b (B, *p* = 0.014 MW). **C** and** D** show that those with continued poor attenders (group 3) performed worse than those with improved attendance (group 4) in both Module 2A and Module 2B (*P* < 0.001 MW)

Groups 1 and 2: In Phase 1b 159 students continued their high attendance whereas 27 students missed two or more events (poorer attendance). There was no difference in marks between these two groups in Module 2A, however, in Module 2B poorer attendees had significantly lower marks than high attendees (*p* = 0.014) suggesting that their performance was adversely affected by the reduced attendance (Fig. 4A and B).

Groups 3 and 4: In Phase 1b, 85 of the students improved their attendance (improved attendees) whereas 81 had continued poor attendance. Analysis of the results in both Phase 1a and Phase 1b showed that the improved attendees performed better than those who maintained poor attendance (Fig. 4).

**Fig. 5:**
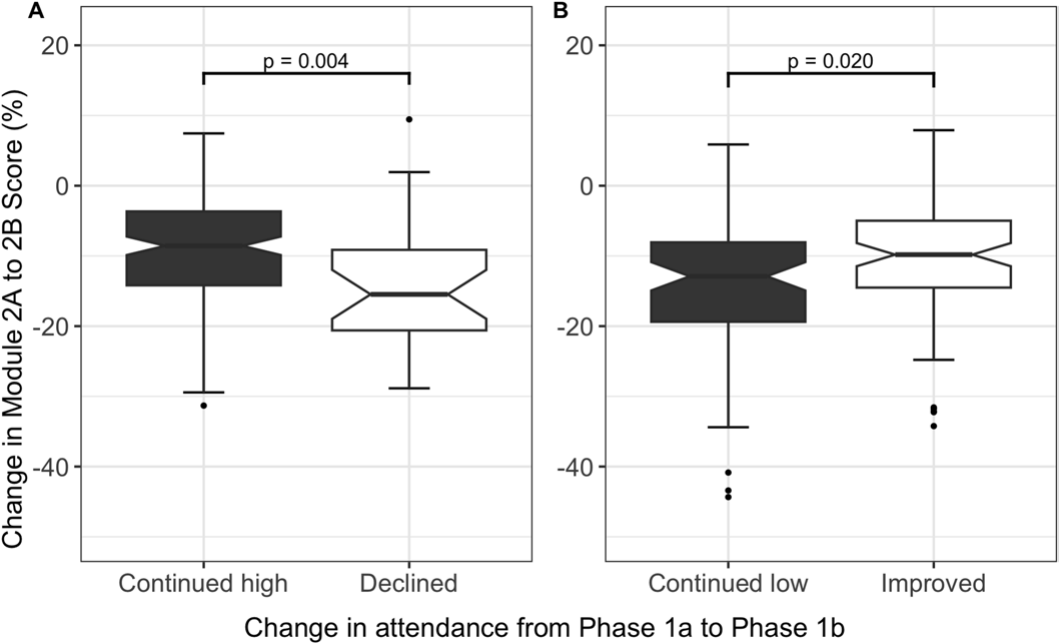
Effect of changes in attendance behaviour on changing exam performance**.** In order to evaluate whether changing attendance behaviour could have an effect on summative results, differences in attendance behaviour were compared to the percentage change in marks from Module 2A to 2B (i.e., from Phase 1a to 1b). **A** shows that poorer attenders performed worse in 2B exams compared to students who maintained high attendance (*p* = 0.004 MW). **B** shows that improved attenders performed better than students who maintained low attendance (*p* = 0.020 MW)

To evaluate whether changing attendance would impact summative performance, the percentage change in marks from Phase 1a to Phase 1b summative exams were calculated and compared. Given that all students had lower marks from Phase 1a to Phase 1b, a “better” performer will have a lower drop in marks. This confirmed that those with good attendance whose attendance became poorer in Phase 1b fared worse in summative exams compared to those who maintained high attendance (*p* = 0.004 MW). It also showed that those whose attendance improved from Phase 1a to Phase 1b performed better than those whose attendance remained poor (*p* = 0.020 MW, Fig. 5).

## Discussion

### Summary of findings

This study shows that attendance at and performance in EFAs and teaching events in the early months of a medicine course can predict the outcome in summative assessment in the same year and highlight students at risk of failure in these summative assessments. Furthermore, students missing relatively small numbers of these assessments (more than 2 out of 10) are at markedly elevated risk of failing summative assessments (up to eightfold). These results are evidence that EFAs are generalisable within the curriculum and therefore align with the second inference of Kane’s validity framework. Furthermore, the stability of results within the secondary analysis of cohort 2 further supports the fulfilment of the generalisability inference.

These assessments and teaching events, therefore, are valid ways for students to monitor their own progress. Furthermore, the data could enable faculty to identify students who are not attending or are performing less well and therefore to intervene to improve study techniques or to help with other underlying issues at an early point in the student’s academic career. Cendán et al. 2018 [35] showed a model that could predict ‘at-risk’ students through plotting examination scores in the first and second year of medical school, with students dropping off “percentile lines”, much like paediatric growth curves. Our data adds to this body of evidence of the ability to predict failure amongst medical students and this work can facilitate the need for early remediation and timely intervention.

Our analysis also shows that students with good early attendance whose attendance falls between the first and second years perform less well than those who maintain attendance between years. Moreover, we showed that not only is worsening attendance over time associated with worse results compared to maintaining good attendance, improving attendance over time is also associated with better results compared to maintaining poor attendance. This provides evidence that students can improve their performance if they improve their attendance from year to year. The fact that high attendance correlates with better performance is unsurprising, but it is reassuring that poor attenders may be able to foster a positive change in their exam performance by improving attendance. In addition, these findings support the implications inference of Kane’s validity framework: firstly, we have evidence that we can make a decision over potential intervention (improving attendance in this instance); secondly, we have evidence that changing these behaviours results in improved performance. Further studies would have to explore whether introducing any interventions to address issues around attendance and engagement could similarly reflect improvement in performance.

Our data are consistent with many other studies [5–12] showing a strong correlation between attendance and summative performance leading to suggestions that attendance should be mandatory. Counter to this suggestion is the need to develop students as independent learners and the loss of student autonomy that would result from such a policy. Furthermore, the effects of mandatory attendance policies may be small and may adversely affect some students who have developed successful independent learning approaches. These students can be seen in the U-shaped relationship identified by a study comparing performance and attendance in second-year medical students [36]. Whether this U-shaped relationship is limited to courses with a heavy bias towards didactic lectures or will also exist in those with a strong active learning component remains to be seen. However, it seems likely that the crude measure of attendance does not capture the range of student behaviour as some students will be present but not engaged. The use of small formative assessments to identify students at risk of poor summative performance and therefore potentially at risk of not progressing has a distinct advantage over pure attendance as a measure for at least three reasons. Firstly, performance in the assessments is less quantised in nature compared to attendance so provides a richer source of information, secondly, it allows students who have decided to work independently to engage with a small number of events and measure the effectiveness of their chosen method of study and finally it is possible to identify those who have attended but still not performed well. These latter students are likely to be those in greatest need of assistance with their study skills. Consistent with this suggestion our analysis shows that students performing in the bottom half of our EFAs were at higher risk of failing summative assessments. This analysis therefore identifies students at risk and therefore in need of additional assistance at an early time-point in their university career.

### Validity of EFAs

When considering the overall validity of EFAs for use as a modified form of programmatic assessment within the curriculum, it is important to consider each aspect of Kane’s framework in turn. Firstly, in terms of scoring, as discussed in the introduction the choice of individual items and assessment instruments was chosen in light of recent research showing the higher reliability, validity and authenticity of VSAQs as an addition to the SBAQs [30–32]. Secondly, in terms of the generalisability of these results, this study has shown that the EFAs are predictive tools of summative performance and correlate with these scores. Moreover, showing this across two cohorts helps to demonstrate that there is reproducibility of these findings and EFAs have stability as predictive tools.

In terms of extrapolation there are two aspects to consider. The first is that in the world of formative assessment, the ‘real-world’ is performance in summative examinations and we have shown here that there is strong correlation between them. The second aspect is whether this also ties in to the wider ‘real-world’ of clinical competency. It is challenging to demonstrate this, however the authors suggest that future work could focus on how EFA performance predicts or correlates with performance in the new medical licensing assessment (MLA) which is being introduced across UK medical schools by the General Medical Council and Medical Schools Council in 2024–5 [37], as well as potentially with the Prescribing and Safety Assessment [38]. The last inference to consider is the implications inference. In the case of EFAs the decision-making process is not around progression, but rather making a decision to intervene at some early stage to address issues with engagement (that is, attendance at, and performance in, both teaching sessions and further formative assessments). We have shown that students who improve their attendance perform better and that students whose attendance declines perform worse, which provides a strong background that such intervention would be beneficial. Nonetheless, future studies could assess whether interventions (e.g., mandatory attendance, additional teaching, or other forms of support) produce measurable and lasting improvement in summative performance.

### Importance of findings

Previous literature, including a recent paper by Dewar et al. [22], have mainly looked at single formatives predicting performance in summative assessments, however in this study we are looking at multiple longitudinal formative assessments,drawing on principles of programmatic assessment [25, 28, 29] with a centrally coordinated and aligned plan, multiple assessment tools over time, information-rich feedback, strong mentoring input, and fostering self-assessment and reflection. The implications of these findings are that they enable early identification of at-risk students for intervention. Future studies can assess whether interventions improve student summative outcomes and progression. For those who cannot roll out programmatic assessments fully due to regulatory constraints and medical licensing assessments, this provides an opportunity for us to draw on the benefits of programmatic assessment without the necessity of making progression decisions.

### Limitations of the study

The study presented has a few limitations that restrict its general applicability. Firstly, the students are medical students at a UK university so may not be representative of UK students studying other subjects or of medical students at other universities with different entrance requirements or age demographics. For example, our students are almost all straight out of secondary school rather than graduate students who have experience in independent learning. Secondly, the teaching and assessment of the students occurred during the COVID-19 pandemic. This has led to changes in both teaching and assessment. The students starting in 2019 were initially taught face to face, on campus with their second year of study delivered predominantly online with some face-to-face teaching. Students starting in 2020 were taught predominantly online with limited face-to-face teaching. All assessments for both sets of students were delivered online and not proctored. Performance in cohort 1 was above what we would normally expect for exams where we had comparable data from the previous curriculum whereas performance in cohort 2 was within the normal range. However, the similarity in the results from the two cohorts suggests that the conclusions are robust.

## Conclusion

This study has provided evidence of validity for EFAs and shown that both attendance and performance in EFAs associate with summative performance. The data also suggest that EFAs have the potential to be an early warning system for both students and faculty. The ability of faculty to identify students at risk raises the potential for them to intervene to improve study skills or provide other forms of support to aid progression. Given the ability to predict early performance, future work could focus on exploring further the generalisability through comparisons with MLA/PSA performance or similar, as well as implications through the evaluation of early interventions. Additionally, further exploration into the risk factors that determine poor early performance and poor attendance can lead to an increased understanding of why certain students struggle and provide the ability to intervene with specific measures.

### Supplementary Information

**Additional file 1:** **Supplementary Table S1. **Correlation of EFA results with overall summative results and individual summative module components in Phase 1a and Phase 1b. **Figure S1. **Formative assessment performance associates with summative exam performance in Phase 1a. **Figure S2. **Formative assessment performance associates with summative exam performance in Phase 1b. **Figure S3. **Effect of missing more than 2 events on score for cohort 1 in Phase 1a. **Figure S4. **Effect of missing more than 2 events on score for cohort 1 in Phase 1b. **Figure S5. **Effect of missing more than 2 events on score for cohort 2 in Phase 1a. 

## Data Availability

The datasets analysed in the current study are not publicly available as they include examination results but are available from the corresponding author on reasonable request.
