# Supplementary material for: The validity of Engagement and Feedback Assessments (EFAs): identifying students at risk of failing
Source: BMC Med Educ. 2023 Nov 15;23:866. doi: 10.1186/s12909-023-04828-7 (PMC10652541; doi:10.1186/s12909-023-04828-7)
Supplement: Supplementary file 1 — Additional file 1: Supplementary Table S1. Correlation of EFA results with overall summative results and individual summative module components in Phase 1a and Phase 1b. Figure S1. Formative assessment performance associates with summative exam performance in Phase 1a. Figure S2. Formative assessment performance associates with summative exam performance in Phase 1b. Figure S3. Effect of missing more than 2 events on score for cohort 1 in Phase 1a. Figure S4. Effect of missing more than 2 events on score for cohort 1 in Phase 1b. Figure S5. Effect of missing more than 2 events on score for cohort 2 in Phase 1a. [file 12909_2023_4828_MOESM1_ESM.docx]

**Supplementary Information - Can attendance and formative metrics identify students at risk of failing summative exams**

**Supplementary results**

*Analysis of Module 4A summative TBLs*

The course included summative assessments in the first term in the form of the Module 4A TBLs which includes the individually answered iRAT components. Given that students are likely to engage with summative assessments, we investigated whether summative iRAT performance gave a better prediction of later summative performance than the EFA assessments. We also determined whether the prediction was improved by combining both EFA and summative assessments undertaken in the first term. Alone, the first term summative iRAT performance was associated with all summative exam performance (first term summative iRAT vs Module 1A: r=0.40, p<0.001, Module 2A: r=0.38, p<0.001, Anatomy spotter: r=0.30, p<0.001, overall weighted score: r=0.46, p<0.001) but the associations were not as strong as those with the first term EFA performance probably due to the larger number of events in the EFA data set. However, when combined with the EFA performance the associations were similar to those obtained using the combined autumn and spring term EFA data (Module 1A: r=0.58, p<0.001, Module 2A main: r=0.57, p<0.001, Anatomy spotter: r=0.43, p<0.001, overall performance r=0.64, p<0.001).

**Supplementary Table S1.** Correlation of EFA results with overall summative results and individual summative module components in Phase 1a and Phase 1b.

| Phase | EFAs used | Exam correlated with | Correlation, r^†^ | p-value^‡^ |
| --- | --- | --- | --- | --- |
| 1a (Year 1) | Terms 1 and 2 | Module 1A | 0.55 | <0.001 |
|  |  | Module 1B | 0.54 | <0.001 |
|  |  | Anatomy spotter | 0.39 | <0.001 |
|  |  | Overall summative | 0.58 | <0.001 |
|  | Term 1 only | Module 1A | 0.50 | <0.001 |
|  |  | Module 1B | 0.49 | <0.001 |
|  |  | Anatomy spotter | 0.35 | <0.001 |
|  |  | Overall summative | 0.53 | <0.001 |
|  |  |  |  |  |
| 1b (Year 2) | Terms 1 and 2 | Module 2B | 0.58 | <0.001 |
|  |  | Module 3B | 0.42 | <0.001 |
|  |  | CPA | 0.47 | <0.001 |
|  |  | Overall summative | 0.58 | <0.001 |
|  | Term 1 only | Module 2B | 0.65 | <0.001 |
|  |  | Module 3B | 0.52 | <0.001 |
|  |  | CPA | 0.38 | <0.001 |
|  |  | Overall summative | 0.57 | <0.001 |
| ^†^Calculated using the Spearman rank correlation coefficient, $\rho$  ^‡^This represents the first term only (i.e., very early performance) | | | | |


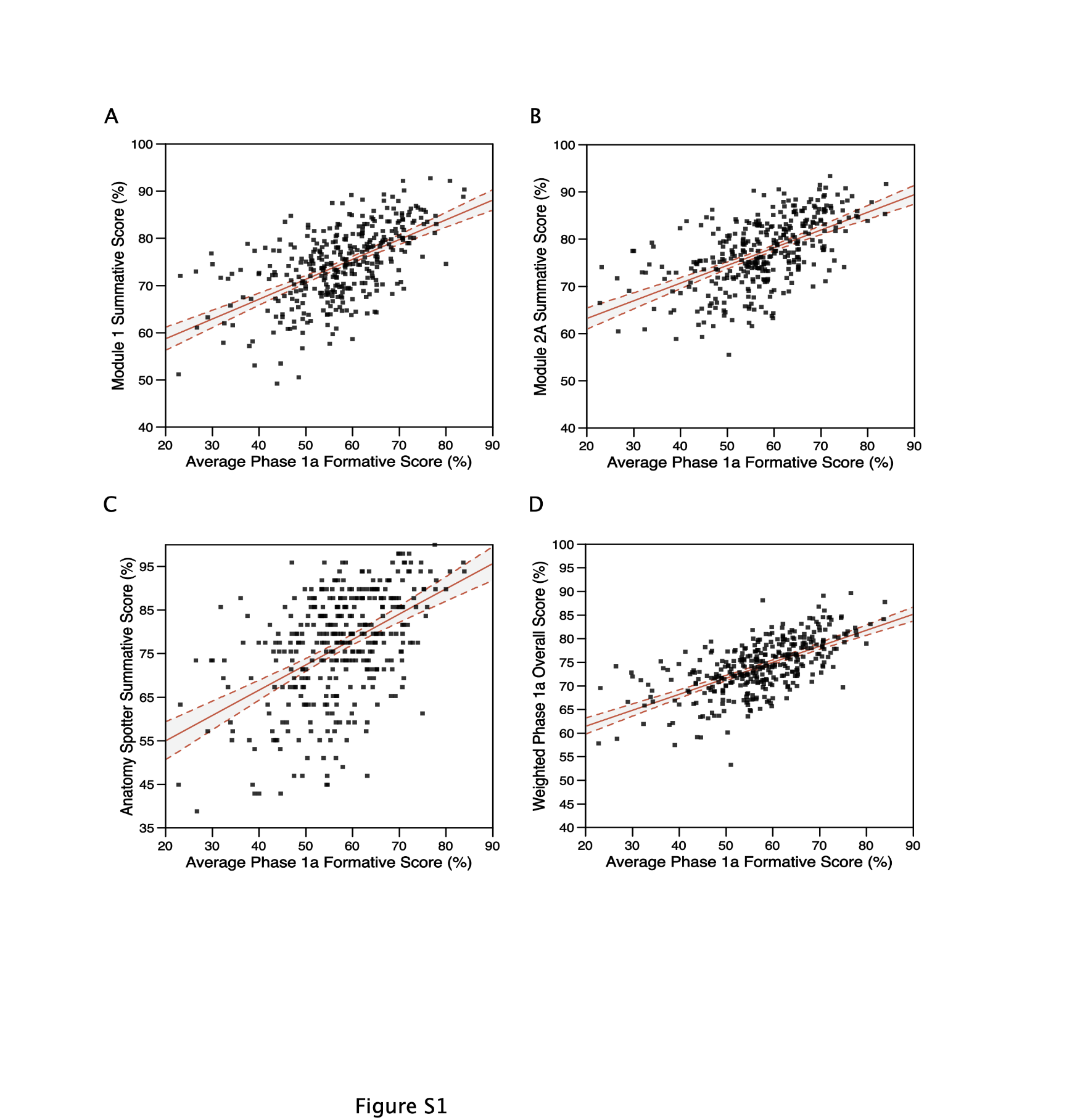
**Supplementary Figure S1**

**Figure S1. Formative assessment performance associates with summative exam performance in Phase 1a**

Average score for all formative attended was correlated with exam performance for cohort in each of the individual papers (A-C) and the weighted overall score (D). In each assessment formative score was positively correlated with summative score (A. Module 1: r=0.58, p<0.001, B. Module 2A main: r=0.54, p<0.001, C. Anatomy spotter: r=0.39, p<0.001, D. Overall performance r=0.58, p<0.001)

**Supplementary Figure S2**


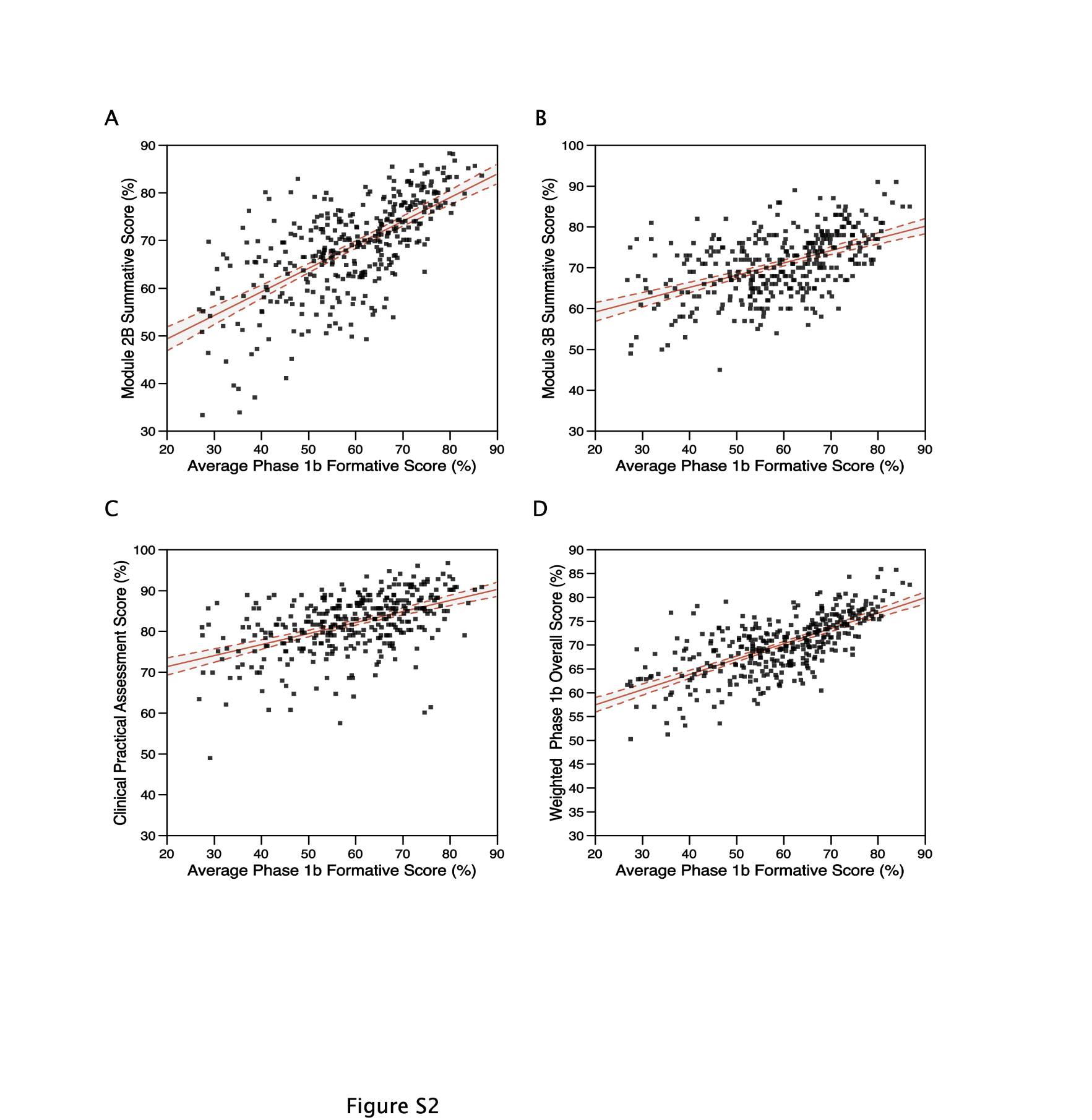


**Figure S2. Formative assessment performance associates with summative exam performance in Phase 1b**

Average score for all formative attended was correlated with exam performance for cohort in each of the individual written papers (A-B), clinical practical assessment (C) and the weighted overall score (D). In each assessment formative score was positively correlated with summative score (A. Module 2B: r=0.58, p<0.001, B. Module 3B: r=0.42 p<0.001, C. CPA: r=0.47, p<0.001, D. overall performance r=0.58, p<0.001)

**Supplementary Figure S3**


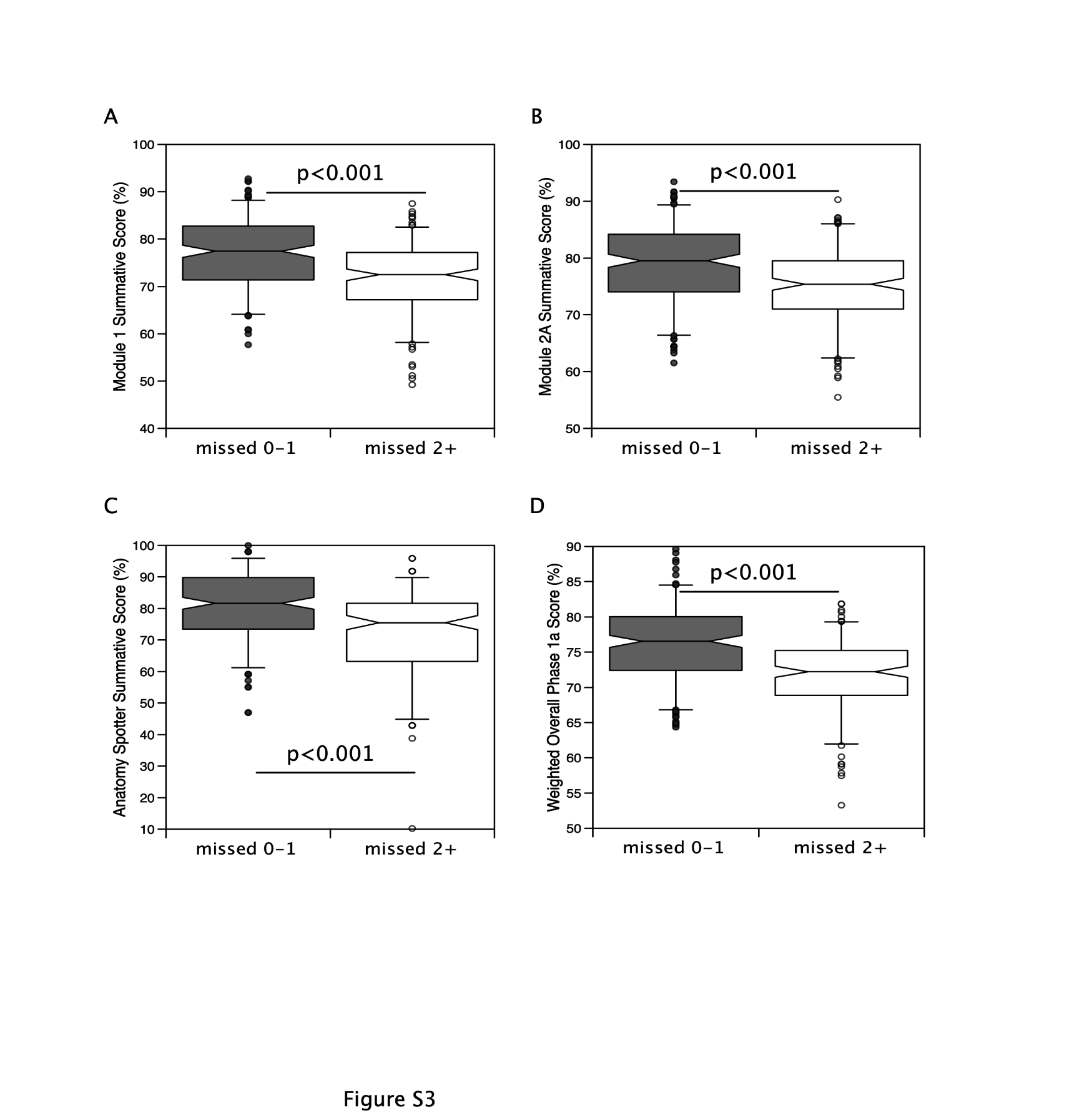


**Figure S3. Effect of missing more than 2 events on score for cohort 1 in Phase 1a.**

Students were grouped by attendance at formative events into those who missed 0 or 1 event and those who missed 2 or more events. Performance in each exam was compared between the two groups and performance of the students with greatest attendance was always higher than those who attended least (P<0.001 MW)

**Supplementary Figure S4**


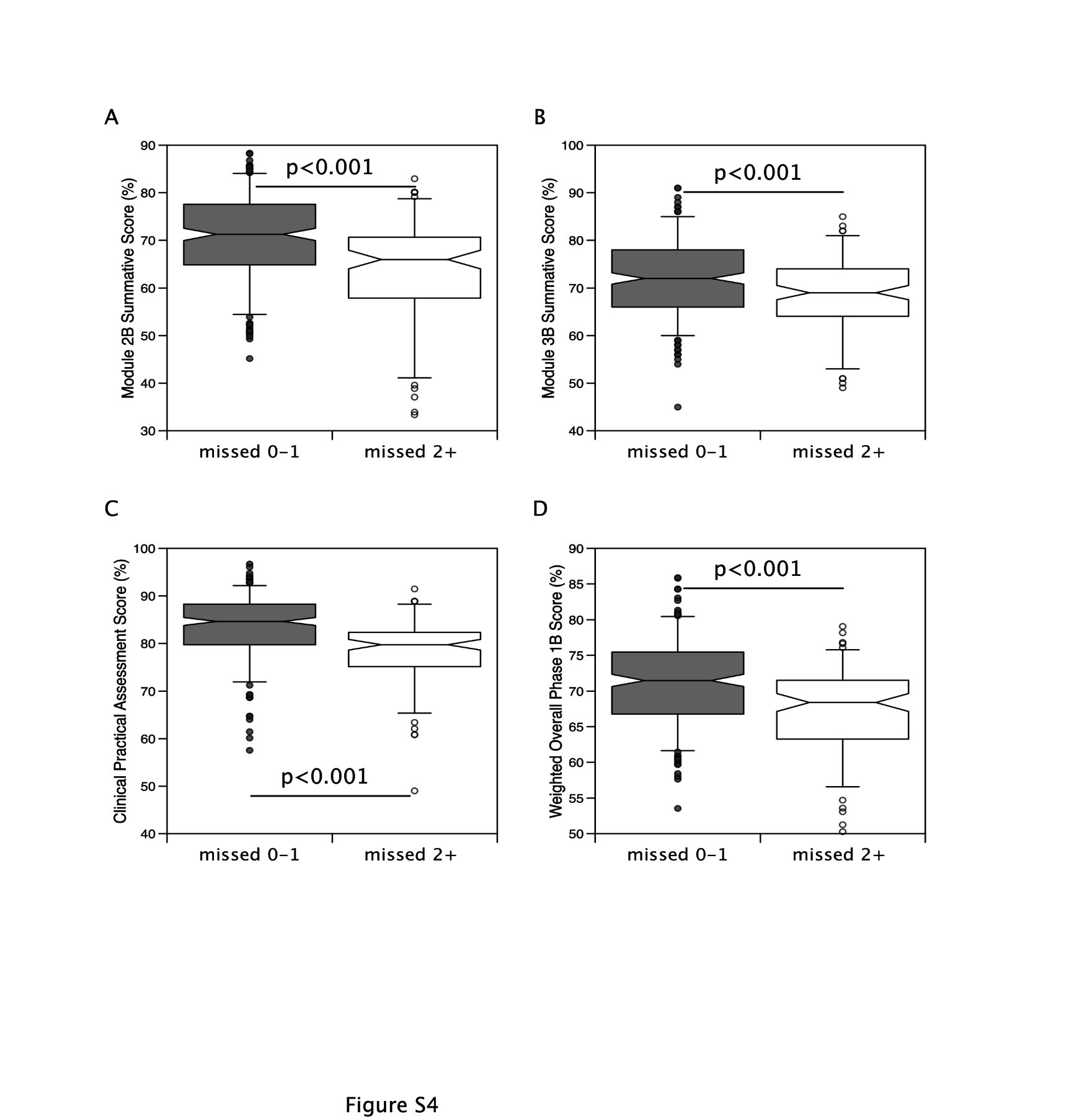


**Figure S4. Effect of missing more than 2 events on score for cohort 1 in Phase 1b.**

Students were grouped by attendance at formative events into those who missed 0 or 1 event and those who missed 2 or more events. Performance in each exam was compared between the two groups and performance of the students with greatest attendance was always higher than those who attended least (P<0.001 MW)

**Supplementary Figure S5**


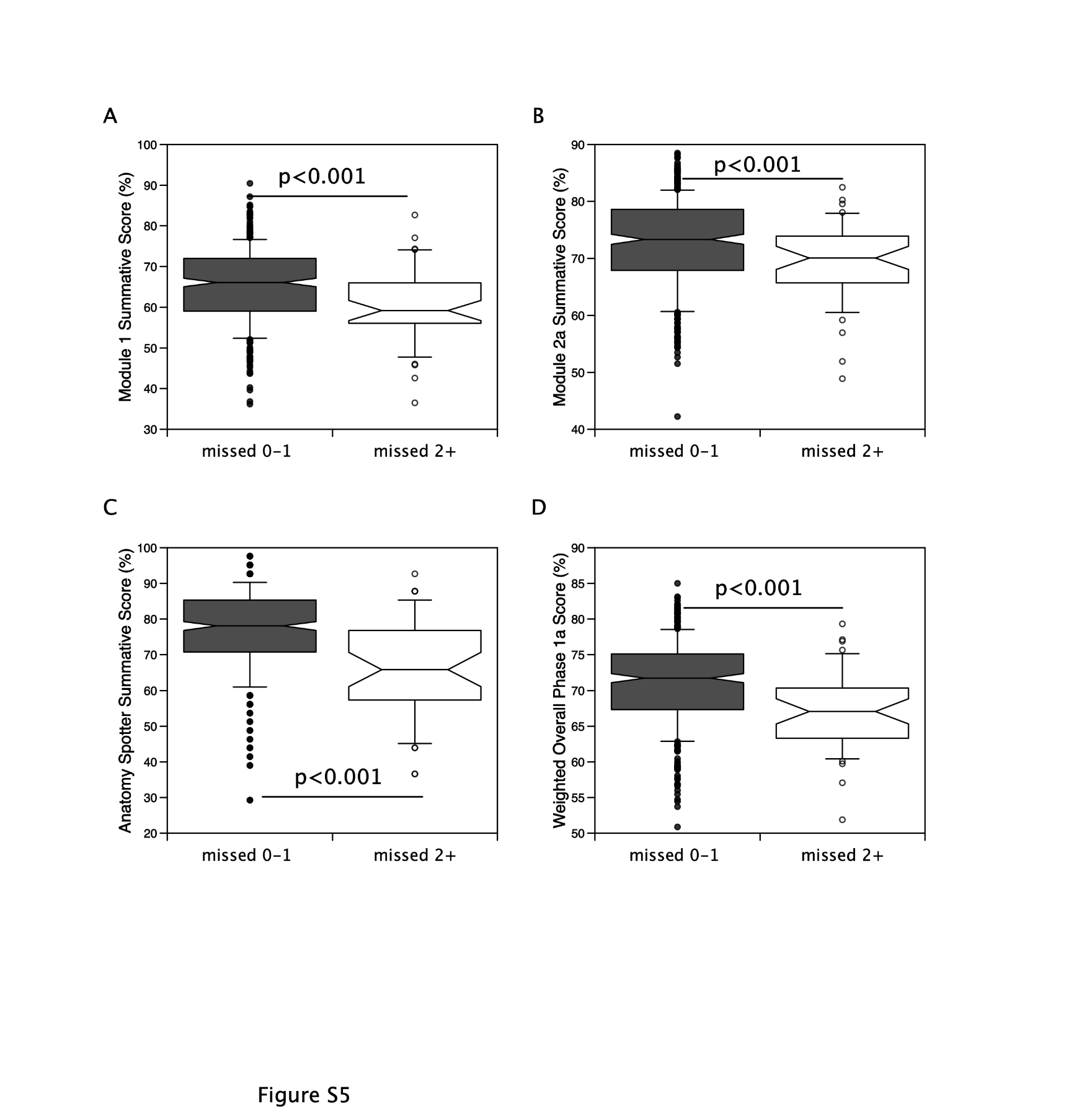


**Figure S5. Effect of missing more than 2 events on score for cohort 2 in Phase 1a.**

Students were grouped by attendance at formative events into those who missed 0 or 1 event and those who missed 2 or more events. Performance in each exam was compared between the two groups and performance of the students with greatest attendance was always higher than those who attended least (P<0.001 MW).
